# Cross-sectional examination of ultra-processed food consumption and adverse mental health symptoms

**DOI:** 10.1017/S1368980022001586

**Published:** 2022-11

**Authors:** Eric M Hecht, Anna Rabil, Euridice Martinez Steele, Gary A Abrams, Deanna Ware, David C Landy, Charles H Hennekens

**Affiliations:** 1 Charles E Schmidt College of Medicine, Florida Atlantic University, 2800 South Ocean Boulevard 3G, Boca Raton, FL 33432, USA; 2 University of Miami, Miller School of Medicine, Miami, FL, USA; 3 Institute of Etiological Research, Boca Raton, FL, USA; 4 University of Sao Paulo, Sao Paulo, Brazil; 5 Prisma Health, Department of Medicine, Division of Gastroenterology and Liver Center, University of South Carolina-SOM, Greenville, SC, USA; 6 Georgetown University Medical Center, Washington, DC, USA; 7 University of Kentucky, College of Medicine, Lexington, KY, USA

**Keywords:** Ultra-processed foods, Mental health, National Health and Nutrition Examination Survey, Epidemiology

## Abstract

**Objective:**

To explore whether individuals who consume higher amounts of ultra-processed food (UPF) have more adverse mental health symptoms.

**Design:**

Using a cross-sectional design, we measured the consumption of UPF as a percentage of total energy intake in kilo-calories using the NOVA food classification system. We explored whether individuals who consume higher amounts of UPF were more likely to report mild depression, more mentally unhealthy days and more anxious days per month using multivariable analyses adjusting for potential confounding variables.

**Setting:**

Representative sample from the United States National Health and Nutrition Examination Survey between 2007 and 2012.

**Participants:**

10 359 adults aged 18+ without a history of cocaine, methamphetamine or heroin use.

**Results:**

After adjusting for covariates, individuals with the highest level of UPF consumption were significantly more likely to report at least mild depression (OR: 1·81; 95 % CI1·09, 3·02), more mentally unhealthy (risk ratio (RR): 1·22; 95 % CI 1·18, 1·25) and more anxious days per month (RR: 1·19; 95 % CI 1·16, 1·23). They were also significantly less likely to report zero mentally unhealthy (OR: 0·60; 95 % CI 0·41, 0·88) or anxious days (OR: 0·65; 95 % CI 0·47, 0·90).

**Conclusions:**

Individuals reporting higher intakes of UPF were significantly more likely to report mild depression, more mentally unhealthy and more anxious days and less likely to report zero mentally unhealthy or anxious days. These data add important information to a growing body of evidence concerning the potential adverse effects of UPF consumption on mental health.

Mental illnesses including depression and anxiety are leading causes of morbidity, disability and mortality^(1,2)^. Dietary patterns may influence mental health. For example, poor dietary patterns which lack essential nutrients, have a high glycaemic index and are high in added sugars may lead to adverse mental health symptoms^(3–6)^. In addition, in animal models, poor diets dysregulate brain insulin which affects mood, decreases neuronal levels of serotonin and dopamine and increases neuroinflammation as measured by inflammatory cytokines^(7–10)^. Poor diets and the consumption of non-nutrient additives in animal models can also adversely affect the intestinal microbiome which, in turn, can lead to systemic and neuroinflammation^(11)^.

The NOVA food classification is a widely used system recently adopted by the Food and Agricultural Organization of the United Nations^(12)^. NOVA considers the nature, extent and purpose of food processing in order to categorise foods and beverages into four groups: unprocessed or minimally processed foods, processed culinary ingredients, processed foods and ultra-processed foods (UPF)^(13,14)^.

UPF are defined as industrial formulations of processed food substances (oils, fats, sugars, starch, protein isolates) that contain little or no whole food and typically include flavourings, colourings, emulsifiers and other cosmetic additives^(15)^. UPF are convenient, low cost, quick to prepare or ready-to-eat preparations of food that result from extensive ‘physical, biological, and chemical processes’ that create food products that are deficient in original and natural food^(16)^. The most commonly consumed UPF include many sugar-sweetened beverages, reconstituted meat products, packaged snacks, chips, breakfast cereals, cookies, cake, chips, and breads and numerous other packaged foods. The ultra-processing of food depletes its nutritional value and also increases the number of calories, as UPF tend to be high in added sugar, saturated fat and salt, while low in protein, fibre, vitamins, minerals and phytochemicals^(17,18)^. Over 70 % of packaged foods in the USA are classified as UPF and represent approximately 60 % of all consumed calories^(19,20)^.

While there is some evidence regarding UPF consumption and depression^(21–23)^, data are sparse regarding other adverse mental health symptoms including anxiety and mentally unhealthy days. In this Research Article, we explored a nationally representative sample of the US population, whether individuals who consume high amounts of UPF report significantly more adverse mental health symptoms including depression, anxiety and mentally unhealthy days.

## Methods

### Data source and participants

The National Health and Nutrition Examination Survey (NHANES) is a series of cross-sectional evaluations of a representative sample of the non-institutionalised population of the USA. NHANES is comprised of four major components, including questions regarding demographics and health, health examination, laboratory testing and a 24-h dietary recall. Further details about NHANES have been described elsewhere^(24,25)^. Using a cross-sectional design, we combined three cycles from NHANES between 2007 and 2012. We included individuals with dietary data and information on mild depression, mentally unhealthy days, anxious days and covariates. We excluded individuals who self-reported the current or past use of cocaine, methamphetamine or heroin because of a lack validation studies using the 9-question Patient Health Questionnaire (PHQ) evaluation to detect mild depression and other mental health symptoms in individuals who use recreational drugs (*n* 2129). The final sample consisted of 10 359 US adults aged 18 years and older.

### Exposure of ultra-processed food

We applied the NOVA classification to all of the recorded United States Department of Agriculture’s Food and Nutrient Database for Dietary Studies (USDA FNDDS) 8-digit Food Codes to the NHANES data. The details of the procedures to classify FNDDS Food Codes according to the NOVA system have been previously described^(26)^. USDA’s FNDDS 2007–2012 were used to code dietary intake data and calculate Food Code energy intakes^(27)^. For homemade recipes, we calculated the underlying ingredient (SR Code) energy values using variables from both FNDDS 2007–2012 and USDA National Nutrient Database for Standard Reference, Legacy Release^(26)^. Using the average of two NHANES 24-h dietary recalls when available (and 1 d otherwise), we quantified each individual’s consumption of UPF in kilo-calories and calculated the percentage energy intake per day, in kilo-calories consumed as UPF. The proportion of respondents with one and two 24-h dietary recall was 10·6 and 89·4 %, respectively. Subjects were categorised according to their UPF consumption into five evenly divided categories. These categories allowed for a sufficiently large reference group (0–19 %) that could act as a proxy to a non-exposed group. The sample sizes for each group based upon % UPF consumption were: 0–19 %, *n* 305; 20–39 %, *n* 1860; 40–59 %, *n* 4023; 60–79 %, *n* 3286; and ≥80 %, *n* 885.

### Outcome: adverse mental health symptoms

We measured three mental health symptoms: (1) mild depression; (2) number of mental unhealthy days and (3) number of anxious days. Symptoms of depression were ascertained from the PHQ-9. The PHQ-9 is a validated and reliable measure for depression. Respondents with a PHQ-9 score of five points or greater were categorised as having symptoms of mild depression^(28)^. The number of mentally unhealthy days was obtained from the response to the question: ‘*During the past 30 d, how many days was your mental health not good?*’ (range: 1–30). This question is a validated measure of mental health and is highly correlated with mental health symptoms^(29)^. The number of anxious days was obtained from the response to the question: ‘*During the past 30 days, how many days did you feel worried, tense, or anxious?’* (range: 1–30). This question is also a validated measure of chronic anxiety^(30)^.

### Covariates

The following available socio-demographic covariates were included in the analysis: (1) gender (man/woman); (2) age (18–29/30–39/40–49/50–59/60–69 years old); (3) race/ethnicity (Mexican/Other Hispanic/non-Hispanic White/non-Hispanic Black/Other Race) and (4) poverty status calculated as a ratio of the monthly family income specific to family size (less than or equal to poverty level/greater than poverty level). The health-related covariates included smoking (never/former/current), exercise (no physical activity: reported no moderate or vigorous activity; less than recommend physical activity: <150 min of moderate or < 75 min of vigorous activity/week and recommended physical activity: ≥150 min of moderate or > 75 min of vigorous activity/week) as well as BMI categorised as underweight (<18·5 kg/m^2^), healthy weight (18·5–24·9 kg/m^2^), overweight (25–29·9 kg/m^2^) and obese (30 kg/m^2^ and above)^(31,32)^.

### Data analysis

Descriptive statistics were generated for each adverse mental health symptom, mild depression, number of mentally unhealthy days and number of anxious days, as well as available covariates using frequency/percentages or medians/interquartile range, where appropriate. For mild depression, we used logistic regression to model the probability of a PHQ-9 score of five or greater which signifies at least mild depression. We then modelled the outcomes of ‘number of mentally unhealthy days’ and ‘number of anxious days’ using zero-inflated Poisson regression^(33)^. The zero-inflated Poisson regression model has two components, count and logit. The count component model generates risk ratios (RR) of reporting more mentally unhealthy or anxious days over the prior 30 d. The logit component model predicts the probability of a zero count of the outcome and reports Odds Ratio (OR). UPF and the covariates were tested independently in unadjusted models and covariates with *P*-values ≤ 0·1 in their respective unadjusted model were included in the final adjusted model. We considered statistical significance to be based on a two-sided *P* value of less than 0·05. All statistical analyses were performed using SAS software (v9.4; SAS Institute, Inc.) and R (R Foundation for Statistical Computing). NHANES sampling and survey weights were used in the analysis.

## Results

### Descriptive statistics

Among the 10 359 respondents, the median age was 42·2 years, 66·2 % were non-Hispanic Whites, 52·9 % were women and 84·6 % had a family poverty ratio greater than the national level. A total of 68·3 % were overweight (32·3 %) or obese (36 %), 61·0 % had never smoked and 45·6 % reported no physical activity. The median UPF consumption as defined by energy intake percentage was 57·1 % with an interquartile range from 44·9 to 68·6 %. Mild depression was reported in 21·3 % of all respondents. The median number of mentally unhealthy and anxious days were 0 (interquartile range: 0·0–3·3) and 1·1 (interquartile range: 0·0–6·0), respectively (Table 1). Distribution of these characteristics by UPF consumption category is presented in Table 1.

**Table 1:** Baseline characteristics of 10 359 adults aged 18+ years in US NHANES 2007 through 2012

	Percentage of calories consumed as ultra-processed foods										Overall *n* 10 359	
	0–19 % *n* 305		20–39 % *n* 1860		40–59 % *n* 4023		60–79 % *n* 3286		≥80 % *n* 885			
	Median	IQR	Median	IQR	Median	IQR	Median	IQR	Median	IQR	Median	IQR
Age	48·1	34·4–59·1	45·4	33·1–57·3	43·7	30·3–56·1	40·5	27·9–52·9	34·3	23·2–47·1	42·2	29·1–54·5
	%	95 % CI	%	95 % CI	%	95 % CI	%	95 % CI	%	95 % CI	%	95 % CI
Gender (women)	45·3	37·4, 53·2	52·7	49·6, 55·7	54·0	52·0, 56·0	52·9	50·5, 55·2	51·5	47·1, 55·9	52·9	51·9, 54·0
Race/ethnicity												
Mexican	4·8	2·4, 7·3	10·4	8·2, 12·6	10·5	7·8, 13·2	7·2	5·0, 9·5	4·2	2·2, 6·1	8·7	6·5, 10·9
Other Hispanic	13·0	7·8, 18·1	10·5	7·1, 13·9	6·1	4·4, 7·8	3·7	2·6, 4·8	3·0	1·6, 4·3	5·9	4·2, 7·6
Non-Hispanic White	45·0	35·0, 55·1	56·2	49·9, 62·5	65·7	61·3, 70·1	71·7	67·0, 76·3	73·1	66·1, 80·1	66·2	61·7, 70·7
Non-Hispanic Black	9·7	6·2, 13·2	10·8	8·5, 13·0	11·4	9·4 , 13·3	13·4	10·2, 16·5	15·3	10·1, 20·5	12·3	9·9, 14·6
Other race	27·4	18·2, 36·6	12·2	9·2, 15·1	6·4	5·1, 7·7	4·1	3·2, 5·0	4·4	2·4, 6·4	6·9	5·8, 8·1
BMI category												
Underweight <18	1·8	0·0, 3·9	2·5	1·6, 3·4	2·0	1·3, 2·8	2·1	1·4, 2·7	3·3	1·7, 4·8	2·2	1·8, 2·6
Healthy 18·5–24·9	38·0	29·9, 46·0	34·5	31·1, 37·8	29·2	26·8, 31·5	26·9	24·6, 29·3	29·1	24·4, 33·8	29·4	27·5, 31·4
Overweight 25–29·9	30·5	24·3, 36·8	35·6	32·2, 38·9	34·0	31·8, 36·3	30·7	28·6, 32·8	27·2	23·7, 30·6	32·3	30·8, 33·9
Obese > 30	29·7	20·7, 38·6	27·5	24·6, 30·5	34·8	32·3, 37·3	40·3	38·0, 42·7	40·5	36·3, 44·7	36	34·3, 37·8
Poverty level or lower	19·4	13·0, 25·7	15·0	13·0, 17·1	13·2	11·4, 15·0	16·2	13·5, 18·8	20·1	14·5, 25·7	15·4	13·5, 17·4
Smoking status												
Current smoker	18·7	12·0, 25·5	14·7	12·5, 17·0	14·6	12·8, 16·5	20·7	18·5, 22·8	25·8	21·7, 29·9	17·9	16·2, 19·6
Former smoker	19·6	12·4, 26·8	21·4	18·2, 24·5	22·9	20·5, 25·3	17·7	15·6, 19·7	16·1	12·3, 20·0	20·2	18·8, 21·6
Never smoker	61·7	50·7, 72·7	63·9	60·6, 67·3	62·5	59·9, 65·0	61·6	58·4, 64·9	58·1	52·8, 63·3	61·9	59·6, 64·2
Physical activity												
No physical activity	48·5	39·3, 57·7	41·3	37·3, 45·3	44·0	41·0, 47·1	47·4	44·4, 50·4	49·0	43·5, 54·5	45·6	43, 48·2
Less than recommended physical activity	20·6	13·3, 28·0	16·7	14·4, 18·9	18·6	16·9, 20·4	19·7	17·6, 21·8	18·1	15·4, 20·8	18·6	17·3, 19·8
Recommended physical activity	30·9	22·3, 39·5	42·0	37·9, 46·2	37·3	34·3, 40·4	32·9	30·0, 35·8	32·9	28·0, 37·9	35·8	33·3, 38·4
	Median	IQR	Median	IQR	Median	IQR	Median	IQR	Median	IQR	Median	IQR
UPF consumption (% of total energy intake)	15·7	11·6–18·0	33·7	28·8–37·1	51·3	46·1–55·4	67·7	63·6–72·9	85·7	82·4–89·8	57·1	44·9, 68·6
Number of anxious days	0·0	0·0–4·0	1·0	0·0–4·7	0·8	0·0–4·9	1·4	0·0–6·6	1·9	0·0–9·3	1·1	0·0–6·0
Number of mentally unhealthy days	0·0	0·0–1·3	0·0	0·0–2·0	0·0	0·0–2·9	0·0	0·0–4·0	0·0	0·0–5·6	0·0	0·0–3·3
	%	95 % CI	%	95 % CI	%	95 % CI	%	95 % CI	%	95 % CI	%	95 % CI
Mild depression (PHQ-9 score of ≥ 5)	17·3	11·6, 23·0	17·8	15·4, 20·2	19·2	17·2 , 21·3	22·9	21·2, 24·7	30·3	26·7, 33·9	21·3	19·9, 22·6

PHQ-9, Patient Health Questionnaire; IQR, interquartile range.

The missing data on outcomes were as follows: depression (*n* 6; 0·06 %), mentally unhealthy days (*n* 16; 0·15 %) and anxious days (*n* 12; 0·11 %). Since less than 10 % of the data were missing for the main outcome, our analyses were conducted without further weight adjustment or imputation to account for missing data^(34)^.

### Association between ultra-processed food consumption and adverse mental health outcomes

**Fig. 1:**
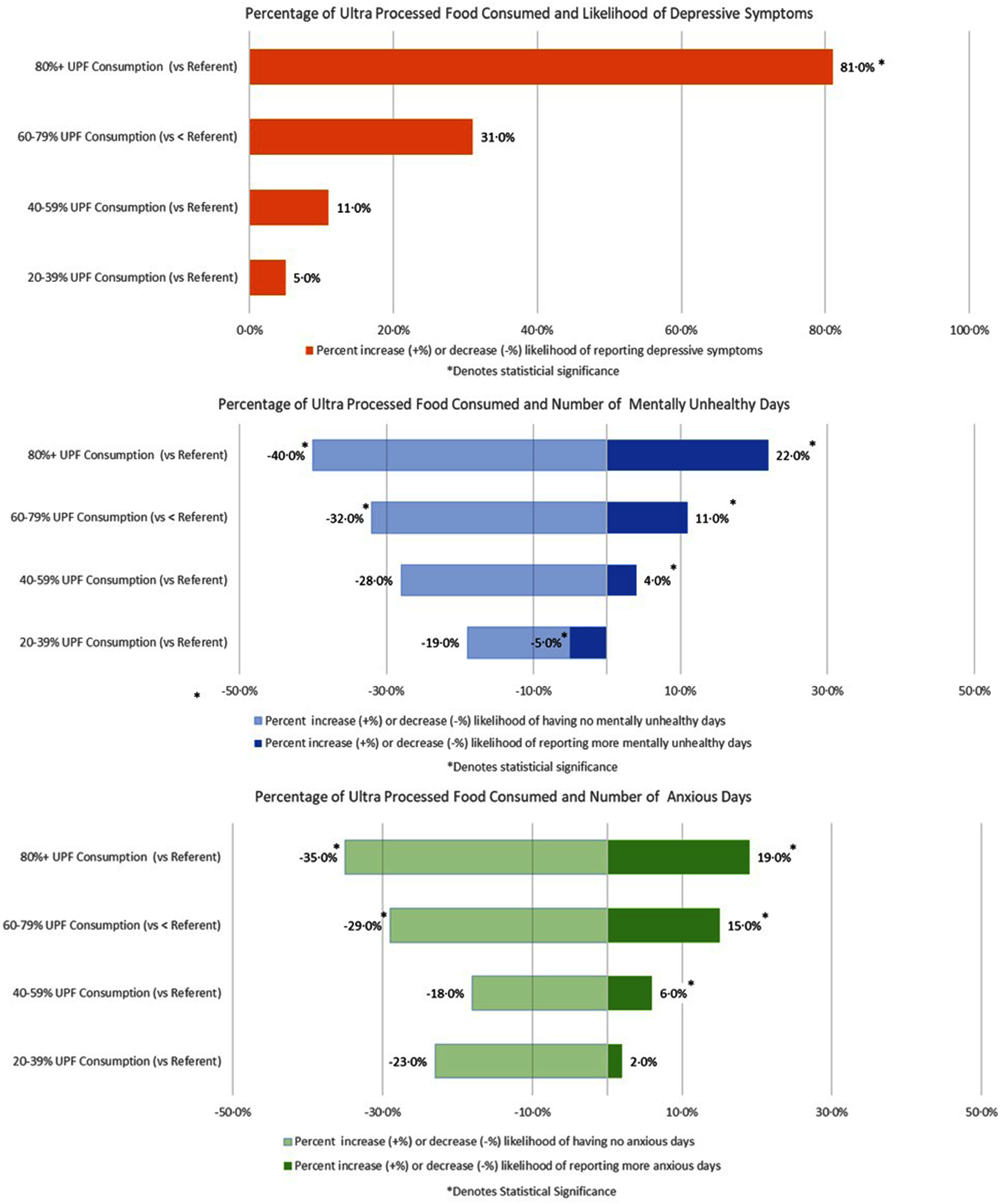
Adjusted percentage likelihood (increase or decrease) of mild depression (OR), number of mentally unhealthy days (RR) and number of anxious days (RR) by category of ultra-processed food consumption with <20 % as the referent level

All models were adjusted for by age, gender, race/ethnicity, BMI, poverty level, smoking status and physical activity. Respondents with the highest *v*. lowest level of UPF consumption had a significantly higher probability of mild depression (OR: 1·81; 95 % CI 1·09, 3·02) (Fig. 1, Table 2) and were significantly more likely to report a higher number of mentally unhealthy days (RR: 1·22; 95 % CI 1·18, 1·25) and anxious days (RR: 1·19; 95 % CI 1·16, 1·23) (Fig. 1, Table 3). For each increasing level of UPF consumption, the RR for each of these outcome measures also significantly increased (Tables 2 and 3).

**Table 2:** Unadjusted and adjusted analyses regarding ultra-processed food exposure, relevant covariates and the outcome of mild depression

	Outcome: mild depression			
	Unadjusted		Adjusted*	
	OR	95 % CI	OR	95 % CI
UPF consumption				
20–39 %	1·04	0·68, 1·58	1·05	0·64, 1·71
40–59 %	1·14	0·77, 1·69	1·11	0·73, 1·69
60–79 %	1·43	0·95, 2·14	1·31	0·84, 2·04
≥ 80 %	2·08**	1·31, 3·29	1·81**	1·09, 3·02
0–19 % (reference)	1·00		1·00	
Age				
30–39 years old	0·98	0·82, 1·18	1·02	0·81, 1·29
40–49 years old	1·16	0·97, 1·39	1·18	0·95, 1·46
50–59 years old	1·03	0·87, 1·23	1·07	0·87, 1·33
60–69 years old	0·80**	0·68, 0·96	0·82	0·65, 1·03
18–29 years old (reference)	1·00		1·00	
Gender				
Women	1·71**	1·53, 1·90	1·75**	1·53, 2·00
Men (reference)	1·00		1·00	
Race/ethnicity				
Mexican	1·12	0·97, 1·29	1·01	0·87, 1·17
Other Hispanic	1·54**	1·27, 1·88	1·41**	1·15, 1·72
Non-Hispanic Black	1·29**	1·11, 1·51	1·10	0·95, 1·29
Other race	0·92	0·72, 1·18	0·92	0·69, 1·24
Non-Hispanic White (reference)	1·00		1·00	
BMI category				
Underweight <18	1·49	1·05, 2·21	1·33	0·89, 1·99
Overweight 25–29·9	0·98	0·82, 1·17	1·05	0·85, 1·29
Obese > 30	1·59**	1·36, 1·87	1·53**	1·30, 1·79
Healthy 18·5–24·9 (reference)	1·00		1·00	
Poverty level				
Lower than poverty level	2·30**	1·91, 2·76	1·91**	1·62, 2·26
Greater than poverty level (reference)	1·00		1·00	
Smoking status				
Current smoker	1·99	1·75, 2·27	1·73**	1·50, 1·99
Former smoker	0·98	0·84, 1·14	1·10	0·93, 1·30
Never smoker	1·00		1·00	
Physical activity				
None	2·25**	1·95, 2·58	1·99**	1·72, 2·30
Less than recommended	1·42**	1·18, 1·70	1·30**	1·12, 1·69
Recommended (reference)	1·00		1·00	

* Adjusted for age, gender, race/ethnicity, BMI category, poverty level, smoking status and physical activity.

** Indicates statistical significance (< 0·05).

**Table 3:** Unadjusted and adjusted risk ratios regarding ultra-processed food exposure, relevant covariates and the outcomes of the number of mentally unhealthy and anxious days self-reported over the prior 30 d

	Outcome: more mentally unhealthy days				Outcome: more of anxious days			
	Unadjusted risk ratio	95 % CI	Adjusted risk ratio*	95 % CI	Unadjusted risk ratio	95 % CI	Adjusted risk ratio*	95 % CI
UPF consumption								
20–39 %	0·91**	0·88, 0·93	0·95**	0·92, 0·98	0·98	0·95, 1·00	1·02	0·98, 1·06
40–59 %	0·97**	0·94, 0·99	1·04**	1·01, 1·06	1·01	0·99, 1·04	1·06**	1·03, 1·10
60–79 %	1·06**	1·03, 1·09	1·11**	1·08, 1·14	1·13**	1·10, 1·15	1·15**	1·12, 1·18
≥ 80 %	1·20**	1·17, 1·23	1·22**	1·18, 1·25	1·20**	1·17, 1·23	1·19**	1·16, 1·23
0–19 % (reference)	1·00		1·00		1·00		1·00	
Age								
30–39 years old	1·08**	1·07, 1·10	1·06**	1·05, 1·07	1·09**	1·08, 1·10	1·09**	1·08, 1·10
40–49 years old	1·14**	1·13, 1·16	1·07**	1·06, 1·08	1·22**	1·21, 1·23	1·17**	1·16, 1·18
50–59 years old	1·28**	1·27, 1·30	1·19**	1·17, 1·20	1·16**	1·15, 1·17	1·12**	1·11, 1·13
60–69 years old	1·11**	1·1, 1·12	1·08**	1·06, 1·09	1·08**	1·07, 1·09	1·04**	1·02, 1·05
18–29 years old (reference)	1·00		1·00		1·00		1·00	
Gender								
Women	1·04**	1·03, 1·05	1·03**	1·02, 1·04	1·09**	1·08, 1·10	1·08**	1·07, 1·09
Men (reference)	1·00		1·00		1·00		1·00	
Race/ethnicity								
Mexican	0·95**	0·94, 0·96	0·99	0·98, 1·00	0·94**	0·93, 0·95	0·93**	0·92, 0·94
Other Hispanic	1·05**	1·04, 1·06	1·05**	1·04, 1·07	1·05**	1·04, 1·06	1·02**	1·01, 1·04
Non-Hispanic Black	1·11**	1·10, 1·12	1·06**	1·05, 1·07	0·98**	0·97, 0·99	0·94**	0·93, 0·95
Other race	1·02**	1·01, 1·04	1·06**	1·04, 1·07	0·92**	0·90, 0·93	0·99	0·97, 1·01
Non-Hispanic White (reference)	1·00		1·00		1·00		1·00	
BMI category								
Underweight <18	1·20**	1·18, 1·23	1·16**	1·14, 1·19	1·17**	1·15, 1·19	1·16**	1·14, 1·19
Overweight 25–29·9	1·09**	1·08, 1·10	1·08**	1·07, 1·10	1·02**	1·01, 1·03	1·01	1·00, 1·03
Obese > 30	1·22**	1·21, 1·23	1·16**	1·14, 1·17	1·14**	1·13, 1·15	1·08**	1·07, 1·09
Healthy 18·5–24·9 (reference)	1·00		1·00		1·00		1·00	
Poverty level								
Lower than poverty level	1·27**	1·26, 1·28	1·16**	1·15, 1·17	1·27**	1·26, 1·29	1·16**	1·15, 1·17
Greater than poverty level (reference)	1·00		1·00		1·00		1·00	
Smoking status								
Current smoker	1·41**	1·40, 1·42	1·35**	1·34, 1·36	1·43**	1·42, 1·43	1·34**	1·34, 1·35
Former smoker	1·12**	1·11, 1·13	1·16**	1·14, 1·17	1·05**	1·04, 1·06	1·09**	1·08, 1·10
Never smoker	1·00		1·00		1·00		1·00	
Physical activity								
None	1·43**	1·42, 1·45	1·26**	1·25, 1·27	0·98	0·97, 1·00	1·24**	1·23, 1·25
Less than recommended	0·97**	0·96, 0·98	0·92**	0·90, 0·93	1·37**	1·36, 1·38	0·94**	0·92, 0·95
Recommended (reference)	1·00		1·00		1·00		1·00	

* Adjusted for age, gender, race/ethnicity, BMI category, poverty level, smoking status and physical activity.

** Indicates statistical significance (< 0·05).

In addition, after adjusting for covariates, respondents with the highest *v*. lowest level of UPF consumption were significantly less likely to report zero mentally unhealthy (OR: 0·60; 95 % CI 0·41, 0·88) and zero anxious days (OR: 0·65; 95 % CI 0·47, 0·90) (Table 4).

**Table 4:** Unadjusted and adjusted OR regarding the likelihood of self-reporting zero mentally unhealthy and anxious days over the prior 30 d as well as relevant covariates, according to the level of ultra-processed food consumption

	Outcome: reporting zero mentally unhealthy days				Outcome: reporting zero of anxious days			
	Unadjusted OR	95 % CI	Adjusted OR*	95 % CI	Unadjusted OR	95 % CI	Adjusted OR*	95 % CI
UPF consumption								
20–39 %	0·76	0·52, 1·10	0·81	0·57, 1·16	0·64**	0·47, 0·87	0·77	0·55, 1·07
40–59 %	0·61**	0·44, 0·86	0·72	0·51, 1·01	0·66**	0·49, 0·87	0·82	0·58, 1·15
60–79 %	0·53**	0·37, 0·76	0·68**	0·48, 0·96	0·56**	0·43, 0·73	0·71**	0·51, 0·99
≥ 80 %	0·44**	0·31, 0·63	0·60**	0·41, 0·88	0·49**	0·38, 0·65	0·65**	0·47, 0·90
0–19 % (reference)	1·00		1·00		1·00		1·00	
Age								
30–39 years old	1·31**	1·14, 1·50	1·25**	1·07, 1·46	0·96	0·86, 1·09	0·94	0·81, 1·09
40–49 years old	1·11	0·97, 1·26	1·10	0·92, 1·31	0·86**	0·76, 0·97	0·86	0·74, 1·02
50–59 years old	1·57**	1·35, 1·81	1·47**	1·18, 1·82	1·05	0·92, 1·20	0·98	0·83, 1·17
60–69 years old	2·09**	1·74, 2·51	2·04**	1·62, 2·57	1·57**	1·38, 1·78	1·49**	1·26, 1·77
18–29 years old (reference)	1·00		1·00		1·00		1·00	
Gender								
Women	0·55**	0·50, 0·61	0·52**	0·46, 0·59	0·58**	0·53, 0·63	0·57**	0·51, 0·63
Men (reference)	1·00		1·00		1·00		1·00	
Race/ethnicity								
Mexican	1·34**	1·17, 1·54	1·43**	1·19, 1·72	1·21**	1·05, 1·39	1·28**	1·07, 1·54
Other Hispanic	0·97	0·83, 1·13	1·00	0·83, 1·20	0·90	0·76, 1·07	0·94	0·76, 1·15
Non-Hispanic Black	1·05	0·94, 1·16	1·17**	1·05, 1·30	1·25**	1·12, 1·41	1·37**	1·21, 1·55
Other race	1·27**	1·04, 1·56	1·27**	1·00, 1·62	1·49**	1·28, 1·73	1·52**	1·27, 1·82
Non-Hispanic White (reference)	1·00		1·00		1·00		1·00	
BMI category								
Underweight <18	1·09	0·78, 1·52	1·24	0·82, 1·87	0·98	0·72, 1·34	1·01	0·69, 1·47
Overweight 25–29·9	1·31**	1·15, 1·48	1·15	0·99, 1·35	1·21**	1·10, 1·33	1·15**	1·02, 1·31
Obese > 30	1·12**	1·01, 1·25	1·02	0·89, 1·17	1·09	0·99, 1·21	1·07	0·97, 1·19
Healthy 18·5–24·9 (reference)	1·00		1·00		1·00		1·00	
Poverty level								
Lower than poverty level	0·70**	0·63, 0·79	0·83**	0·73, 0·93	0·84**	0·77, 0·91	0·87**	0·78, 0·96
Greater than poverty level (reference)	1·00		1·00		1·00		1·00	
Smoking status								
Current smoker	0·70**	0·61, 0·79	0·71**	0·60, 0·85	0·96	0·86, 1·06	0·94	0·83, 1·07
Former smoker	1·12	0·97, 1·31	0·92	0·78, 1·09	1·23**	1·08, 1·40	1·15	0·98, 1·36
Never smoker	1·00		1·00		1·00		1·00	
Physical activity								
None	0·89**	0·80, 0·99	0·90	0·79, 1·03	0·88**	0·78, 0·99	1·05	0·95, 1·16
Less than recommended	0·81**	0·70, 0·95	0·84**	0·71, 0·98	1·00	0·92, 1·09	0·94	0·82, 1·08
Recommended (reference)	1·00		1·00		1·00		1·00	

* Adjusted for age, gender, race/ethnicity, BMI category, poverty level, smoking status and physical activity.

** Indicates statistical significance (< 0·05).

## Discussion

In this nationally representative sample of American adults, UPF constituted 57 % of total energetic intake. Individuals who consumed the most UPF as compared with those who consumed the least amount had statistically significant increases in the adverse mental health symptoms of mild depression, ‘mentally unhealthy days’ and ‘anxious days’. They also had significantly lower rates of reporting zero ‘mentally unhealthy days’ and zero ‘anxious days’.

Our data are supported by existing evidence from basic research and other descriptive and observational studies. For example, basic research provides support for the hypothesis that food additives in UPF including emulsifiers and artificial sweeteners can lead to pathophysiological changes that have been associated with mental health symptoms including impaired glucose tolerance, increases in inflammatory mediators, oxidative stress, neuroinflammation, pathogenic changes to neuronal mitochondrial function, as well as alterations in both tryptophan metabolism, and the HPA axis, and changes in the local expression of neurotrophic growth factors^(35)^. Several investigations, including two large prospective cohort studies in Europe, suggest that individuals whose diets lack essential nutrients, have a high glycaemic index, and are high in added sugars also have significantly increased risks of depression and anxiety. They also found that those who consume diets, high in fish, vegetables, olive oil, beans, nuts, PUFA and low in saturated fats, such as the Mediterranean diet, have significantly lower risks of depression^(4–6,9,21,22,36–43)^.

Several meta-analyses of observational studies are compatible with the current findings. In one meta-analysis of twenty observational studies, individuals who consumed diets that included a higher intake of fruit, vegetables, fish and whole grains had lower risks of depression^(4)^. In another, individuals who adhered to the Mediterranean diet had significantly lower rates of depression. In a third meta-analysis, individuals who consumed a diet lower in PUFA and *n*-3 fatty acids reported significantly more mild depression or social anxiety^(44)^. Finally, in one randomised trial, which provides the most reliable evidence for small to moderate effects, those assigned to a 3-month healthy dietary intervention reported significant decreases in moderate-to-severe depression^(43)^.

Our data also suggest that those who consume high levels of UPF consumption also experience significantly more ‘mentally unhealthy’ and ‘anxious’ days and their corresponding decrease in ‘zero mentally unhealthy days’ and ‘zero anxious days’. In another study of elderly adults, those who consumed a poor diet quality as measured by the HEI also had significantly more mentally unhealthy days^(45)^. To the best of our knowledge, there are no data regarding the higher consumption of UPF and the mental health outcomes of ‘zero anxious days’ and ‘zero mentally unhealthy days’.

This original research has several unique strengths. With respect to exposure, the use of the NOVA to classify dietary data allowed determining the level of food processing according to objective and standardised criteria. With respect to outcomes, we utilised three validated measures of adverse mental health symptoms. In addition, the NHANES database is a large and representative sample of the US population. This suggests that the findings are generalisable to the entire USA as well as other Western countries with similar UPF intakes.

This study also has several limitations. In addition to the descriptive study design, other limitations include the self-report of both exposure and outcomes which could result in misclassification of one or both of these measures. Dietary data obtained by 24-h recalls may suffer from recall or social desirability bias; however, the data acquisition method employed by NHANES has been shown to produce accurate intake estimates suitable for assessing population averages^(35,46–48)^. An additional limitation is that NHANES does not consistently collect all of the information needed to assess food processing (i.e. place of meals, product brands)^(49)^. Nevertheless, such misclassification is more likely to be non-differential underestimating the true effect. In addition, while we attempted to control for the potential confounding effects of the available variables, residual confounding is possible especially because lifestyle risk factors tend to cluster^(50)^. We also calculated the proportion of UPF in the diet by using ‘energy ratio’ rather than ‘weight ratio’ which does not properly capture ‘energy devoid’ UPF (e.g. artificially sweetened beverages) and non-nutritional factors related to food processing such as alteration of the food matrix, neo-formed contaminants or food additives. Our study findings are also limited in generalisability to milder grades of depression. Despite these limitations, we believe the most plausible interpretation of these data are to add to the growing body of evidence that individuals who consume higher amounts of UPF have significantly more adverse mental health symptoms.

In summary, these data indicate that individuals with higher intakes of UPF report significantly more mild depression, as well as more mentally unhealthy and anxious days per month, and less zero mentally unhealthy or anxious days per month. When considering these data in the context of the totality of evidence, it can be hypothesised that a diet high in UPF provides an unfavourable combination of biologically active food additives with low essential nutrient content which together have an adverse effect on mental health symptoms. While further research is needed, especially randomised clinical trials, these data add important and relevant information to a growing body of evidence concerning the adverse effects of UPF consumption on mental health symptoms. Since UPF represent the majority of calories consumed by the US population, these data may also have significant clinical and public health implications.

## References

[1] Patel V , Chisholm D , Parikh R et al. (2016) Addressing the burden of mental, neurological, and substance use disorders: key messages from disease control priorities. Lancet 387, 1672–1685. 26454360 10.1016/S0140-6736(15)00390-6

[2] Rehm J & Shield KD (2019) Global burden of disease and the impact of mental and addictive disorders. Curr Psychiatry Rep 21, 10. 30729322 10.1007/s11920-019-0997-0

[3] Rahe C , Unrath M & Berger K (2014) Dietary patterns and the risk of depression in adults: a systematic review of observational studies. Eur J Nutr 53, 997–1013. 24468939 10.1007/s00394-014-0652-9

[4] Lai JS , Hiles S , Bisquera A et al. (2014) A systematic review and meta-analysis of dietary patterns and depression in community-dwelling adults. Am J Clin Nutr 99, 181–197. 24196402 10.3945/ajcn.113.069880

[5] Li Y , Lv MR , Wei YJ et al. (2017) Dietary patterns and depression risk: a meta-analysis. Psychiatry Res 253, 373–382. 28431261 10.1016/j.psychres.2017.04.020

[6] Molendijk M , Molero P , Ortuño Sánchez-Pedreño F et al. (2018) Diet quality and depression risk: a systematic review and dose-response meta-analysis of prospective studies. J Affect Disord 226, 346–354. 29031185 10.1016/j.jad.2017.09.022

[7] Kaplan BJ , Rucklidge JJ , Romijn A et al. (2015) The emerging field of nutritional mental health: inflammation, the microbiome, oxidative stress, and mitochondrial function. Clin Psychol Sci 3, 964–980.

[8] Wang J , Zhou Y , Chen K et al. (2018) Dietary inflammatory index and depression: a meta-analysis. Public Health Nutr 22, 1–7. 10.1017/S1368980018002628 PMC10260661 30319085

[9] Gómez-Pinilla F (2008) Brain foods: the effects of nutrients on brain function. Nat Rev Neurosci 9, 568–578. 18568016 10.1038/nrn2421 PMC2805706

[10] National Center for Chronic Disease Prevention and Health Promotion (2021) Poor Nutrition. https://www.cdc.gov/chronicdisease/resources/publications/factsheets/nutrition.htm (accessed March 2021).

[11] Melo HM , Santos LE & Ferreira ST (2019) Diet-derived fatty acids, brain inflammation, and mental health. Front Neurosci 13, 265. 30983955 10.3389/fnins.2019.00265 PMC6448040

[12] Monteiro CA , Cannon G , Lawrence M et al. (2019) Ultra-Processed Foods, Diet Quality, and Health Using the NOVA Classification System. Rome: FAO.

[13] Monteiro CA , Levy RB , Claro RM et al. (2010) A new classification of foods based on the extent and purpose of their processing. Cad Saude Publica 26, 2039–2049. 21180977 10.1590/s0102-311x2010001100005

[14] Moubarac JC , Parra DC , Cannon G et al. (2014) Food classification systems based on food processing: significance and implications for policies and actions: a systematic literature review and assessment. Curr Obes Rep 3, 256–272. 26626606 10.1007/s13679-014-0092-0

[15] Monteiro CA , Cannon G , Levy RB et al. (2019) Ultra-processed foods: what they are and how to identify them. Public Health Nutr 22, 936–941. 30744710 10.1017/S1368980018003762 PMC10260459

[16] Monteiro CA , Cannon G , Moubarac JC et al. (2018) The UN decade of nutrition, the NOVA food classification and the trouble with ultra-processing. Public Health Nutr 21, 5–17. 28322183 10.1017/S1368980017000234 PMC10261019

[17] Martínez Steele E , Popkin BM , Swinburn B et al. (2017) The share of ultra-processed foods and the overall nutritional quality of diets in the US: evidence from a nationally representative cross-sectional study. Popul Health Metr 15, 6. 28193285 10.1186/s12963-017-0119-3 PMC5307821

[18] Martínez Steele E & Monteiro CA (2017) Association between dietary share of ultra-processed foods and urinary concentrations of phytoestrogens in the US. Nutrients 9, 209. 28264475 10.3390/nu9030209 PMC5372872

[19] Martínez Steele E , Baraldi LG , Louzada ML et al. (2016) Ultra-processed foods and added sugars in the US diet: evidence from a nationally representative cross-sectional study. BMJ Open 6, e009892. 10.1136/bmjopen-2015-009892 PMC4785287 26962035

[20] Baldridge AS , Huffman MD , Taylor F et al. (2019) The healthfulness of the US packaged food and beverage supply: a cross-sectional study. Nutrients 11, 1704. 31344845 10.3390/nu11081704 PMC6722673

[21] Adjibade M , Julia C , Allès B et al. (2019) Prospective association between ultra-processed food consumption and incident depressive symptoms in the French NutriNet-Santé cohort. BMC Med 17, 78. 30982472 10.1186/s12916-019-1312-y PMC6463641

[22] Gómez-Donoso C , Sánchez-Villegas A , Martínez-González MA et al. (2020) Ultra-processed food consumption and the incidence of depression in a Mediterranean cohort: the SUN project. Eur J Nutr 59, 1093–1103. 31055621 10.1007/s00394-019-01970-1

[23] Zheng L , Sun J , Yu X et al. (2020) Ultra-processed food is positively associated with depressive symptoms among United States adults. Front Nutr 7, 600449. 33385006 10.3389/fnut.2020.600449 PMC7770142

[24] Curtin LR , Mohadjer LK , Dohrmann SM et al. (2013) National Health and Nutrition Examination Survey: sample design, 2007–2010. Vital Health Stat 2 160, 1–23. 25090039

[25] Johnson CL , Dohrmann SM , Burt VL et al. (2014) National Health and Nutrition Examination Survey: sample design, 2011–2014. Vital Health Stat 2 162, 1–33. 25569458

[26] USDA & Agricultural Research Service (2018) USDA National Nutrient Database for Standard Reference, Legacy Release. Beltsville, MD: United States Department of Agriculture, Agricultural Research Service.

[27] Montville JB , Ahuja JK , Martin CL et al. (2013) USDA food and nutrient database for dietary studies (FNDDS), 5.0. Proc Food Sci 2, 99–112.

[28] Kroenke K , Spitzer RL & Williams JB (2001) The PHQ-9: validity of a brief depression severity measure. J Gen Intern Med 16, 606–613. 11556941 10.1046/j.1525-1497.2001.016009606.x PMC1495268

[29] Thompson WW , Zack MM , Krahn GL et al. (2012) Health-related quality of life among older adults with and without functional limitations. Am J Public Health 102, 496–502. 22390514 10.2105/AJPH.2011.300500 PMC3487675

[30] Strine TW , Kroenke K , Dhingra S et al. (2009) The associations between depression, health-related quality of life, social support, life satisfaction, and disability in community-dwelling US adults. J Nerv Ment Dis 197, 61–64. 19155812 10.1097/NMD.0b013e3181924ad8

[31] Centers for Disease Control and Prevention (2014) Defining Adult Overweight and Obesity. https://www.cdc.gov/obesity/adult/defining.html (accessed March 2021).

[32] U.S. Department of Health and Human Services (2018) Physical Activity Guidelines Advisory Committee Scientific Report. Washington, DC: U.S. Department of Health and Human Services.

[33] Liu Y , Tian GL , Tang ML et al. (2019) A new multivariate zero-adjusted Poisson model with applications to biomedicine. Biom J 61, 1340–1370. 29799138 10.1002/bimj.201700144

[34] Centers for Disease Control and Prevention (2021) Module 1: Datasets and Documentation 2021. https://wwwn.cdc.gov/nchs/nhanes/tutorials/module1.aspx (accessed March 2021).

[35] Boulangé CL , Neves AL , Chilloux J et al. (2016) Impact of the gut microbiota on inflammation, obesity, and metabolic disease. Genome Med 8, 42. 27098727 10.1186/s13073-016-0303-2 PMC4839080

[36] Oddy WH , Robinson M , Ambrosini GL et al. (2009) The association between dietary patterns and mental health in early adolescence. Prev Med 49, 39–44. 19467256 10.1016/j.ypmed.2009.05.009

[37] Parletta N , Zarnowiecki D , Cho J et al. (2019) A Mediterranean-style dietary intervention supplemented with fish oil improves diet quality and mental health in people with depression: a randomized controlled trial (HELFIMED). Nutr Neurosci 22, 474–487. 29215971 10.1080/1028415X.2017.1411320

[38] Spencer SJ , Korosi A , Layé S et al. (2017) Food for thought: how nutrition impacts cognition and emotion. NPJ Sci Food 1, 7. 31304249 10.1038/s41538-017-0008-y PMC6550267

[39] Gangwisch JE , Hale L , Garcia L et al. (2015) High glycemic index diet as a risk factor for depression: analyses from the women’s health initiative. Am J Clin Nutr 102, 454–463. 26109579 10.3945/ajcn.114.103846 PMC4515860

[40] Knüppel A , Shipley MJ , Llewellyn CH et al. (2017) Sugar intake from sweet food and beverages, common mental disorder and depression: prospective findings from the Whitehall II study. Sci Rep 7, 6287. 28751637 10.1038/s41598-017-05649-7 PMC5532289

[41] Stevens AJ , Rucklidge JJ & Kennedy MA (2018) Epigenetics, nutrition and mental health. Is there a relationship? Nutr Neurosci 21, 602–613. 28553986 10.1080/1028415X.2017.1331524

[42] El Ansari W , Adetunji H & Oskrochi R (2014) Food and mental health: relationship between food and perceived stress and depressive symptoms among university students in the United Kingdom. Cent Eur J Public Health 22, 90–97. 25230537 10.21101/cejph.a3941

[43] Jacka FN , O’Neil A , Opie R et al. (2017) A randomised controlled trial of dietary improvement for adults with major depression (the ‘SMILES’ trial). BMC Med 15, 23. 28137247 10.1186/s12916-017-0791-y PMC5282719

[44] Lin PY , Huang SY & Su KP (2010) A meta-analytic review of polyunsaturated fatty acid compositions in patients with depression. Biol Psychiatry 68, 140–147. 20452573 10.1016/j.biopsych.2010.03.018

[45] Xu F , Cohen SA , Lofgren IE et al. (2018) Relationship between diet quality, physical activity and health-related quality of life in older adults: findings from 2007–2014 national health and nutrition examination survey. J Nutr Health Aging 22, 1072–1079. 30379305 10.1007/s12603-018-1050-4 PMC12275597

[46] Moshfegh AJ , Rhodes DG , Baer DJ et al. (2008) The US department of agriculture automated multiple-pass method reduces bias in the collection of energy intakes. Am J Clin Nutr 88, 324–332. 18689367 10.1093/ajcn/88.2.324

[47] Blanton CA , Moshfegh AJ , Baer DJ et al. (2006) The USDA automated multiple-pass method accurately estimates group total energy and nutrient intake. J Nutr 136, 2594–2599. 16988132 10.1093/jn/136.10.2594

[48] Rumpler WV , Kramer M , Rhodes DG et al. (2008) Identifying sources of reporting error using measured food intake. Eur J Clin Nutr 62, 544–552. 17426745 10.1038/sj.ejcn.1602742

[49] Slining MM , Yoon EF , Davis J et al. (2015) An approach to monitor food and nutrition from “factory to fork”. J Acad Nutr Diet 115, 40–49. 25441958 10.1016/j.jand.2014.09.002 PMC4276507

[50] Schuit AJ , van Loon AJ , Tijhuis M et al. (2002) Clustering of lifestyle risk factors in a general adult population. Prev Med 35, 219–224. 12202063 10.1006/pmed.2002.1064

